# Self-Perception of Aging and Frailty Phenotype Among HIV+ and HIV- Older Men

**DOI:** 10.1093/geroni/igaa057.3008

**Published:** 2020-12-16

**Authors:** Michael Plankey, Karen Nieves-Lugo, Deanna Ware, Sabina Haberlen, Keri Althoff, James Egan, Andre Brown, Mackey Friedman

**Affiliations:** 1 Georgetown University Medical Center, Washington, District of Columbia, United States; 2 National Institutes of Health, Bethesda, Maryland, United States; 3 Johns Hopkins University, Baltimore, Maryland, United States; 4 University of Pittsburgh, Pittsburgh, Pennsylvania, United States

## Abstract

Self-perception of aging is an important predictor of health. We examined the relationship of self-perception of aging (age discrepancy and aging satisfaction) with frailty phenotype between HIV+ and HIV- men in the Multicenter AIDS Cohort Study. 499 HIV+ and 549 HIV- men were included in the analytic sample (median age 61 years IQR 56-66 years). Frailty status was based on the Fried frailty phenotype and measured at semi-annual study visits beginning 3/2015 or 9/2015, 3/2016 and 3/2019. Baseline frailty was: HIV- 8.9%; HIV+ 13.9%. Low aging satisfaction and feeling older was positively associated with remaining frail (ORs: 6.64;95%CI:3.88-11.38; 5.68; 95%CI:3.06-10.56) or transitioning between non-frail and frail states (ORs: 2.72;95%CI:1.56-4.74; 2.50; 95%CI:1.11-5.64),), over a 3-year period. There was no statistically significant difference by HIV status. Assessment of self-perception of aging may be useful in the setting of frailty evaluation among HIV+ and HIV- men.

